# A comprehensive study on electroencephalography and magnetoencephalography sensitivity to cortical and subcortical sources

**DOI:** 10.1002/hbm.25272

**Published:** 2020-11-06

**Authors:** Maria Carla Piastra, Andreas Nüßing, Johannes Vorwerk, Maureen Clerc, Christian Engwer, Carsten H. Wolters

**Affiliations:** ^1^ Institute for Biomagnetism and Biosignalanalysis University of Münster Münster Germany; ^2^ Institute for Computational and Applied Mathematics University of Münster Münster Germany; ^3^ Cognitive Neuroscience, Donders Institute for Brain, Cognition and Behaviour, Radboud University Nijmegen Medical Center Nijmegen The Netherlands; ^4^ Institute of Electrical and Biomedical Engineering, University for Health Sciences Medical Informatics and Technology Hall in Tirol Austria; ^5^ Inria Sophia Antipolis‐Mediterranée Biot France; ^6^ Université Côte d'Azur Nice France; ^7^ Cluster of Excellence EXC 1003, Cells in Motion, CiM, University of Münster Münster Germany; ^8^ Otto Creutzfeldt Center for Cognitive and Behavioral Neuroscience, University of Münster Münster Germany

**Keywords:** electroencephalography, finite element method, magnetoencephalography, sensitivity map, signal‐to‐noise ratio, subcortical sources, volume conduction modeling

## Abstract

Signal‐to‐noise ratio (SNR) maps are a good way to visualize electroencephalography (EEG) and magnetoencephalography (MEG) sensitivity. SNR maps extend the knowledge about the modulation of EEG and MEG signals by source locations and orientations and can therefore help to better understand and interpret measured signals as well as source reconstruction results thereof. Our work has two main objectives. First, we investigated the accuracy and reliability of EEG and MEG finite element method (FEM)‐based sensitivity maps for three different head models, namely an isotropic three and four‐compartment and an anisotropic six‐compartment head model. As a result, we found that ignoring the cerebrospinal fluid leads to an overestimation of EEG SNR values. Second, we examined and compared EEG and MEG SNR mappings for both cortical and subcortical sources and their modulation by source location and orientation. Our results for cortical sources show that EEG sensitivity is higher for radial and deep sources and MEG for tangential ones, which are the majority of sources. As to the subcortical sources, we found that deep sources with sufficient tangential source orientation are recordable by the MEG. Our work, which represents the first comprehensive study where cortical and subcortical sources are considered in highly detailed FEM‐based EEG and MEG SNR mappings, sheds a new light on the sensitivity of EEG and MEG and might influence the decision of brain researchers or clinicians in their choice of the best modality for their experiment or diagnostics, respectively.

## Graphical abstract

Signal‐to‐noise ratio (SNR) maps are a good way to visualize electroencephalography (EEG) and magnetoencephalography (MEG) sensitivity. In our work, we computed EEG and MEG sensitivity maps in a six‐compartment anisotropic head model. We show that ignoring the cerebrospinal fluid leads to an overestimation of EEG SNR values. For cortical sources, we found that radial and deep are more visible to EEG, tangential (the majority) more to MEG. Finally, our results show that MEG is not fully insensitive to subcortical sources.

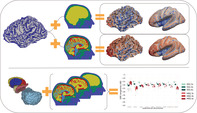

## 1 INTRODUCTION

Electroencephalography (EEG) and magnetoencephalography (MEG) are techniques used to investigate brain activity (Brette & Destexhe, 2012; Hämäläinen, Hari, Ilmoniemi, Knuutila, & Lounasmaa, 1993). EEG and MEG detect the electric potential distribution and the magnetic field, respectively, generated by current sources in the brain, with a unique time resolution. Even if generated by the same sources, MEG and EEG signals differ and carry complementary information (Dassios, Fokas, & Hadjiloizi, 2007). A simple example is the spherical approximation of the head volume conductor model, where (quasi‐)analytical solutions exist for the so‐called EEG (de Munck & Peters, 1993) or MEG (Sarvas, 1987) forward problems, resp., that is, the simulation of the electric potential or the magnetic field at EEG or MEG sensors for a given dipolar current source in the brain. The analytical solution for the MEG shows that radial sources do not produce a magnetic field outside the spherical model, while this is not the case on the EEG side. As a consequence, for deeper sources, the magnetic field does not only get weaker due to the source‐to‐sensor distance, like the EEG also does, but also due to the increasing radial source component. While the main features are preserved, the situation gets, however, more sophisticated in case a realistically shaped head volume conductor model is adopted (Ahlfors, Han, Belliveau, & Hämäläinen, 2010). Moreover, there are studies where the sensitivity of both EEG and MEG to even more realistic head models is shown (Vorwerk et al., 2014).

Signal‐to‐noise ratio (SNR) maps provide a good estimate of EEG and MEG sensitivity to source location and orientation (Goldenholz et al., 2009; Hillebrand & Barnes, 2002). They are informative tools which allow for a correct interpretation of neuroscientific and neurodiagnostic applications such as EEG/MEG source reconstruction or, in a reciprocal sense (Vallaghé, Papadopoulo, & Clerc, 2008; Wagner, Lucka, et al., 2016; Wagner, Burger, & Wolters, 2016), also transcranial electric (TES)/magnetic stimulation (TMS) (Miranda, Lomarev, & Hallett, 2006; Rampersad et al., 2014; Saturnino, Thielscher, Madsen, Knösche, & Weise, 2019) and sensor placement optimization (Guler et al., 2016; Sadleir, Vannorsdall, Schretlen, & Gordon, 2012; Wagner, Burger, & Wolters, 2016). Moreover, SNR maps might guide the choice of preprocessing procedures to apply to record EEG and MEG signals (Bigdely‐Shamlo, Mullen, Kothe, Su, & Robbins, 2015; Goldenholz et al., 2009; Marinkovic, Cox, Reid, & Halgren, 2004).

Necessary ingredients to compute SNR maps are realistic and accurate forward problem solutions. There are different ways to solve the forward problem, for example, analytical formulas (de Munck & Peters, 1993; Ilmoniemi, 1995; Mosher, Leahy, & Lewis, 1999; Sarvas, 1987; Zhang, 1995), boundary element methods (BEMs) (Akalin‐Acar & Gençer, 2004; Fuchs, Wagner, & Kastner, 2001; Kybic, Clerc, Faugeras, Keriven, & Papadopoulo, 2005; Makarov et al., 2020; Oostenveld & Oostendorp, 2002; Stenroos & Sarvas, 2012), finite difference methods (Cuartas Morales, Acosta‐Medina, Castellanos‐Domínguez, & Mantini, 2019; Montes‐Restrepo et al., 2014; Turovets, Poolman, Salman, Malony, & Tucker, 2008), and finite element methods (FEMs) (Bertrand, Thevenet, & Perrin, 1991; Marin, Guerin, Baillet, Garnero, & Meunier, 1998; Miinalainen et al., 2019; Piastra et al., 2018; Pursiainen, Vorwerk, & Wolters, 2016; Schimpf, Ramon, & Haueisen, 2002). In this work, we use FEM because of its ability to approximate the geometric and conductive properties of the head tissue in more detail.

EEG and MEG sensitivities and expected SNR values have been already examined in literature (de Jongh, de Munck, Gonçalves, & Ossenblok, 2005; Fuchs et al., 1998; Goldenholz et al., 2009; Haueisen, Funke, Güllmar, & Eichardt, 2012; Hillebrand & Barnes, 2002; Hunold, Funke, Eichardt, Stenroos, & Haueisen, 2016; Tarkiainen, Liljeström, Seppä, & Salmelin, 2003). In particular, in Goldenholz et al. (2009), EEG and MEG SNR mappings for cortical sources extracted from MRI anatomical information have been computed and visualized, applying BEM in a three‐compartment isotropic head model. In our study, we expand and extend the work of Goldenholz et al. (2009) providing a first comprehensive study where we computed EEG and MEG SNR maps for both cortical and subcortical sources in highly detailed FEM head models, where the cerebrospinal fluid (CSF), gray matter, and the white matter anisotropy is modeled and where the skull compact bone is distinguished from the skull spongy bone. Our dual goal was to investigate the reliability of such sensitivity maps and then analyze the sensitivity results given by such mappings.

In order to assess the level of detail of the head model needed to achieve reliable SNR maps, we computed and compared EEG and MEG SNR maps using FEM in three different head models with increasing level of detail. Starting from an isotropic three‐compartment head model (3CI), where scalp, skull and brain are considered, we increased the number of tissue compartments with an isotropic four‐compartment head model (4CI), where the CSF compartment is added to the three previous ones, until we used a six‐compartment head model (6CA), where both the skull and the brain compartments are further segmented into skull compacta and spongiosa and gray and anisotropic white matter, respectively.

Furthermore, we investigated EEG and MEG sensitivity maps for both cortical and subcortical sources and their modulation by source location, orientation, and depth. The difference between EEG and MEG SNR values are visualized on the cortical surface, and SNR values for both cortical and subcortical sources are represented and compared via boxplots.

## 2 MATERIALS AND METHODS

**FIGURE 1:**
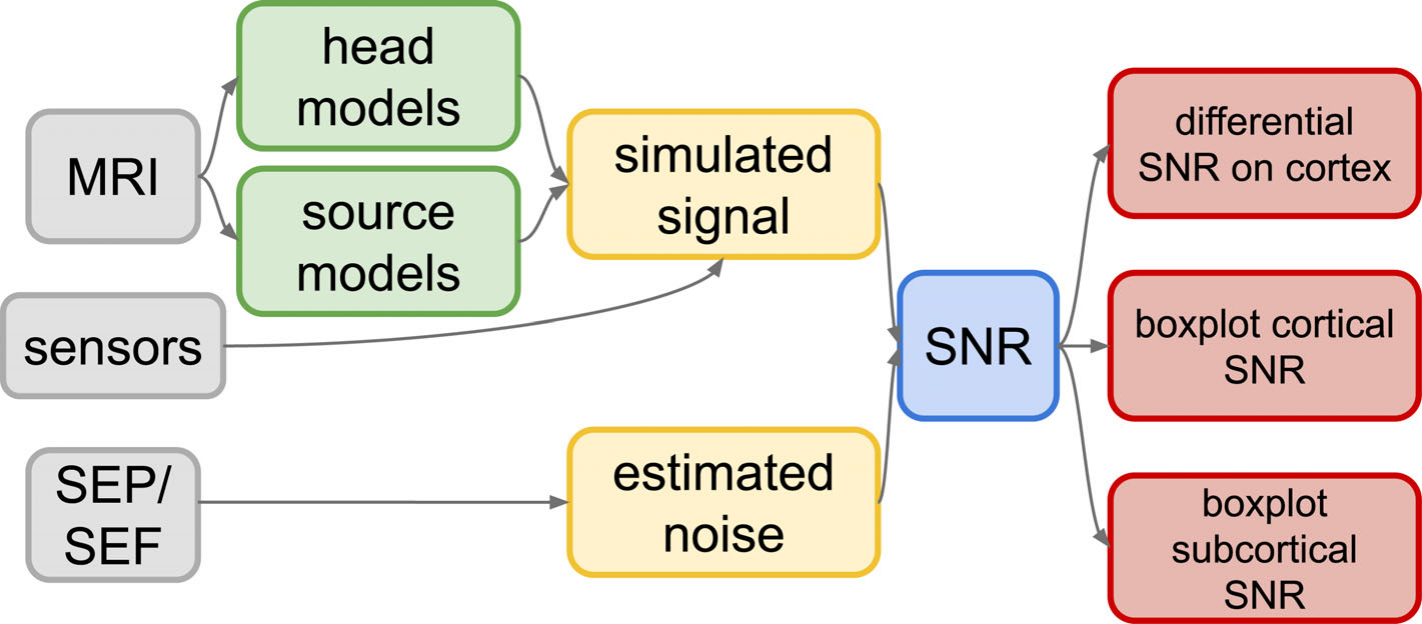
Sketch of the flowchart with the main steps for calculating the signal‐to‐noise ratio (SNR) values in our study. Gray boxes: input data, that is, MRI, somatosensory evoked potentials (SEP) and fields (SEF) and sensor setup; green boxes: MRI‐based models needed as input to the finite element method (FEM) computation; yellow boxes: electroencephalography (EEG) and magnetoencephalography (MEG) signal simulation with FEM and noise estimation from real data; blue box: SNR computation following Formula (1); red boxes: visualization of results, that is, EEG and MEG SNR values for cortical and subcortical sources

The approach adopted in our study is summarized in the flowchart in Figure 1.

### 2.1 MRI data acquisition

A 3 T scanner (MAGNETOM Prisma 3.0 T, Release D13 [Siemens Medical Solutions, Erlangen, Germany]) was used for the acquisition of MRI datasets. We measured a 3D‐T1‐weighted (T1w) fast gradient‐echo pulse sequence (TFE) using water selective excitation to avoid fat shift (TR/TE/FA = 2,300/3.51 ms/8*°*, inversion prepulse with TI = 1.1 s, cubic voxels of 1 mm edge length); 3D‐T2‐weighted (T2w) turbo spin echo pulse sequence (TR/TE/FA = 3,200/408 ms/90*°*, cubic voxels, 1 mm edge length) and DTI using an echo planar imaging sequence (TR/TE/FA = 9,500/79 ms/90*°*, cubic voxels, 1.89 mm edge length), with one volume with diffusion sensitivity *b* = 0 s/mm^2^ (i.e., flat diffusion gradient) and 20 volumes with *b* = 1,000 s/mm^2^ in different directions, equally distributed on a sphere. Another volume with flat diffusion gradient, but with reversed spatial encoding gradients was acquired and used for susceptibility artifact correction (Holland, Kuperman, & Dale, 2010; Ruthotto et al., 2012). During T1w‐MRI measurement, gadolinium markers were placed at the nasion, left and right distal outer ear canal positions for landmark‐based registration of MEG/EEG to MRI. All EEG/MEG and MRI measurements were done in supine position to reduce head movements, to prevent erroneous CSF effects due to brain shift when combining EEG/MEG and MRI (Rice, Rorden, Little, & Parra, 2013) and to stabilize the baseline of the brain activity (Thibault, Lifshitz, & Raz, 2016).

### 2.2 Acquisition of somatosensory‐evoked potential and somatosensory‐evoked field data

Somatosensory‐evoked potential (SEP) and field (SEF) data were simultaneously acquired in a magnetically shielded room using 80 AgCl sintered ring electrodes (EASYCAP GmbH, Herrsching, Germany, 74 EEG channels plus additional six channels to detect eye movements) and a whole‐head MEG system with 275 axial gradiometers and 29 reference sensors (OMEGA2005, VSM MedTech Ltd., Canada). For the detection of cardiac activity, electrocardiography was additionally measured. The MEG reference coils were used to calculate first‐order synthetic gradiometers in order to reduce the interference of magnetic fields originating from distant locations. Prior to the measurements, the electrode positions of the EEG cap were digitized using a Polhemus device (FASTRAK, Polhemus Incorporated, Colchester, VT). Moreover, during the acquisition, the head position inside the MEG was tracked via three head localization coils placed on the nasion, and the left and right distal outer ear canal.

**FIGURE 2:**
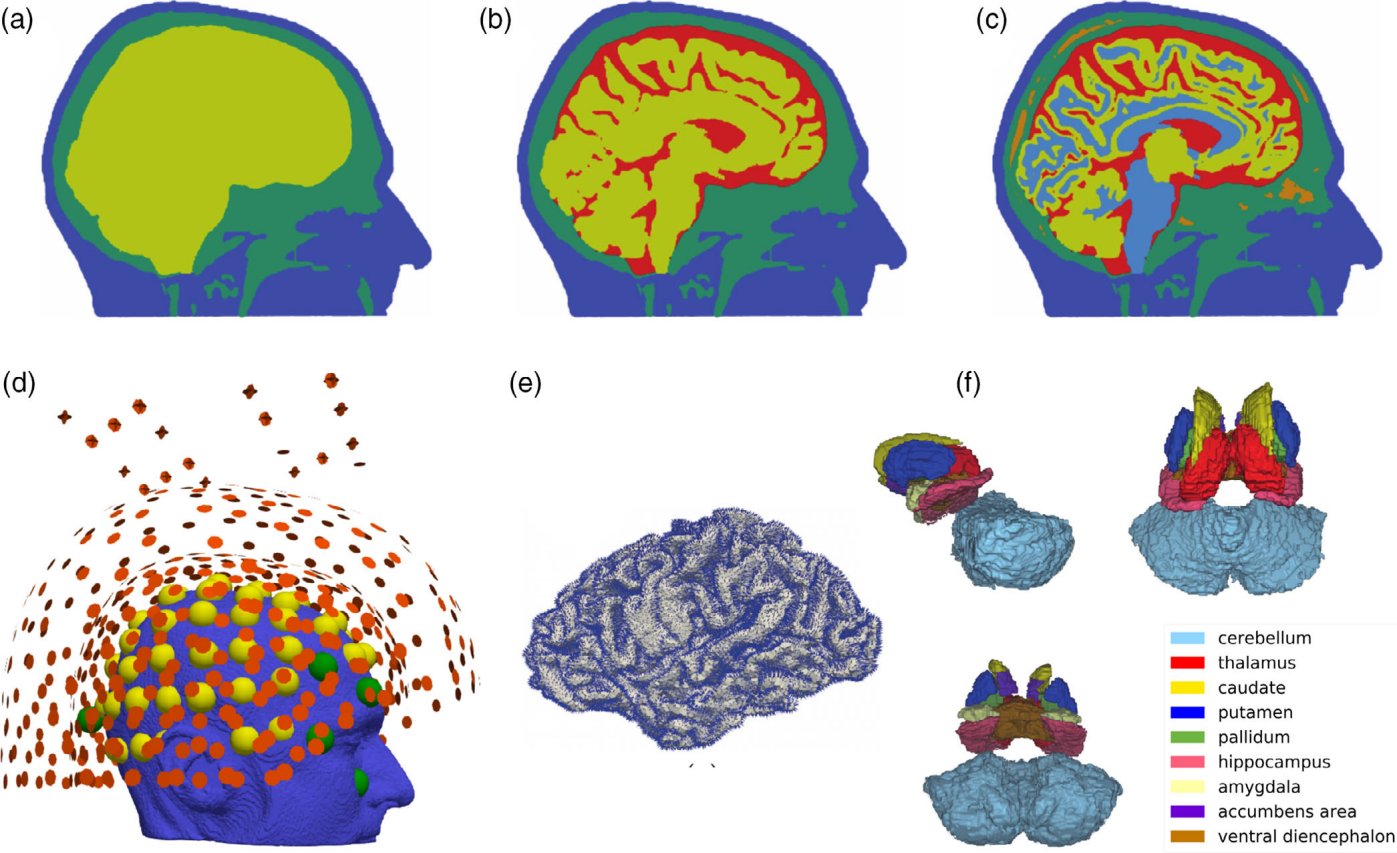
Setup for forward computations. In (a)–(c), the three head models used in the computation of the forward model solutions are shown. In the (3CI) head model, in (a), the skin is depicted in blue, the skull in green and the brain in yellow; in the (4CI) head model, in (b), the additional CSF compartment is colored in red; in the (6CA) head model, in (c), the skull spongiosa is depicted in orange and the white matter in light blue. In (d), the electroencephalography (EEG) and magnetoencephalography (MEG) sensor configurations are shown, that is, 71 electrodes (in yellow), 9 electrodes associated to disregarded channels (in green), and 271 gradiometers (in red) with reference coils for noise cancelation. In particular, we removed the EEG channels labeled as P7, AF8, O9, LO2, SO2, IO2, LO1, SO1, IO1, electrocardiography. In (e), the cortical dipole positions and orientations used in the simulations are shown. In (f), we visualize the nine subcortical structure masks segmented via Freesurfer and visualized in Seg3d in sagittal and in transverse view from above and from below. For every subcortical dipole position, we considered the three Cartesian orientations

At the time of the recording, only 271 gradiometers were active, in addition, as shown in Figure 2d, 45 reference coils were used for noise cancelation. The neurophysiological data are available on the Zenodo portal (Piastra et al., 2020).

### 2.3 Head models

The three images (T1w‐, T2w‐MRI, and DTI) were co‐registered and resampled so that the voxels of the anatomical data are cubic. This last step facilitates the segmentation procedure. Furthermore, the images were cut sufficiently below the skull of the participant, following the suggestions in Lanfer et al. (2012). Subsequently, the segmentation of T1w and T2w was performed in order to separate six volumetric masks representing the six compartments we included in the most realistic model, that is, (6CA). The anisotropic conductivity tensors were deduced from the DTI, following the procedure described in Tuch, Wedeen, Dale, George, and Belliveau (2001) and Aydin et al. (2017). The brain compartment was segmented via the *FreeSurfer* software (http://freesurfer.net/) and the remaining preprocessing and volumetric masks creation was entirely performed via routines available in FieldTrip (Oostenveld, Fries, Maris, & Schoffelen, 2011). In particular, the scalp and skull compartments were segmented via the *spm12* software ((Penny, Friston, Ashburner, Kiebel, & Nichols, 2011); https://www.fil.ion.ucl.ac.uk/spm/software/spm12/), embedded in FieldTrip. The *Seg3d* software (CIBC, 2016) was utilized for an easier visualization of both sliced volumetric masks and automatically generated surfaces, and for quickly checking the output of the segmentation. Once the masks were assembled, a volumetric tetrahedral mesh was created using the *CGAL* software ((The CGAL Project, 2013); https://www.cgal.org) embedded in iso2mesh (Fang & Boas, 2009), resulting in 885,214 nodes and 5,335,615 tetrahedrons. Three head models were constructed and utilized in this study: a three‐compartment isotropic head model (3CI), where scalp, skull and brain are included, a four‐compartment isotropic head model (4CI) where the CSF is additionally distinguished, and a more detailed head volume conductor model with six compartments (6CA), that is, scalp, skull compacta, skull spongiosa, CSF, gray matter, and anisotropic white matter. Specific features of the three models are gathered in Table 1.

**TABLE 1:** Conductivity values (in S/m) of the three models created and used for the sensitivity study: 6CA, six‐compartment head model with anisotropic white matter; 4CI, four‐compartment isotropic head model and 3CI, three‐compartment isotropic head model. The column (:) indicates when the compartment has been split, for example, skull compartment divided between skull compacta and skull spongiosa; while the dash (−) indicates that the compartment has been neglected in the head model. Note that the white matter anisotropic value (i.e., 0.14 S/m) refers to the mean value of the tensor that fits the conductivity value of the isotropic white matter (Vorwerk et al., 2014)

Tissue	6CA (S/m)	4CI (S/m)	3CI (S/m)	
White matter	0.14	—	—	Ramon, Schimpf, Haueisen, Holmes, and Ishimaru (2004)
Gray matter	0.33	—	—	Ramon et al. (2004)
Brain	:	0.33	0.33	Ramon et al. (2004)
CSF	1.79	1.79	—	Baumann, Wozny, Kelly, and Meno (1997)
Skull compacta	0.008	—	—	Dannhauer, Lanfer, Wolters, and Knösche (2011)
Skull spongiosa	0.025	—	—	Dannhauer et al. (2011)
Skull	:	0.01	0.01	Dannhauer et al. (2011)
Scalp	0.43	0.43	0.43	Dannhauer et al. (2011), Ramon et al. (2004)

Abbreviation: CSF, cerebrospinal fluid.

For all three head models, that is, 3CI, 4CI, and 6CA, only the tissue labels were modified accordingly, while the mesh remained the same, since the geometrical error was not studied in this work. In Figure 2a–d, the three models and the EEG/MEG sensors are visualized. The three volumetric meshes are available on the Zenodo portal (Piastra et al., 2020).

### 2.4 Source spaces

In this study, two different source spaces were considered: a cortical surface and a subcortical volume. In both cases, we modeled the sources as point‐like dipolar sources (de Munck, van Dijk, & Spekreijse, 1988; Murakami & Okada, 2006).

With regard to the former, the surface representation of the white matter given by *Freesurfer* was considered. Nodes lying on the gray/white matter surface were projected into the centroids of the closest elements belonging to the gray matter (Euclidean distance was used to compute the closest elements), and considered as dipole positions. The dipole orientations were chosen as the normals of the white matter surface (the normals were computed with the MeshLab toolbox [http://www.meshlab.net/]). This procedure results in several dipoles having the same location but different orientation.

The volumetric subcortical dipolar space was created by extracting a subcortical volumetric mask (erosion of 1 voxel) from the *Freesurfer* parcellation, which identified nine subcortical regions: cerebellum, thalamus, caudate, putamen, pallidum, hippocampus, amygdala, accumbens area, and ventral diencephalon. Subsequently, a tetrahedral volumetric mesh was constructed with iso2mesh for each of the nine subcortical regions identified by *Freesurfer*. The nodes of each mesh were considered as dipole positions. Modeling the orientation of subcortical dipolar sources is not as trivial as modeling cortical dipole orientations. The neural generators of deep structures can be classified in *open* and *closed* field cells, according to the resulting electromagnetic field produced by their dendritic arborization (Attal, Maess, Friederici, & David, 2012). In the first group, there is a preferred orientation of the neural architecture, in the second group there is not. According to this fact, and following (Attal et al., 2012) and (Krishnaswamy et al., 2017), in this study, we considered the three Cartesian components for each mesh node as source orientations. More details on the subcortical areas and number of dipoles considered for each area can be found in Table 2.

**TABLE 2:** Subcortical areas included in the study with respective number of dipoles employed as subcortical source space

Name	#Dipoles
Cerebellum	73,509
Thalamus	10,815
Caudate	3,846
Putamen	5,592
Pallidum	3,042
Hippocampus	5,805
Amygdala	2,451
Accumbens area	1,065
Ventral diencephalon	5,778

In Figure 2e,f, the cortical dipole positions and orientations used in the simulations and the volumetric subcortical masks are visualized. A total of 278,621 cortical dipoles with normal orientations and 111,903 subcortical dipoles with Cartesian orientations were utilized for this study.

#### 2.4.1 Depth and orientation estimation of cortical sources

**FIGURE 3:**
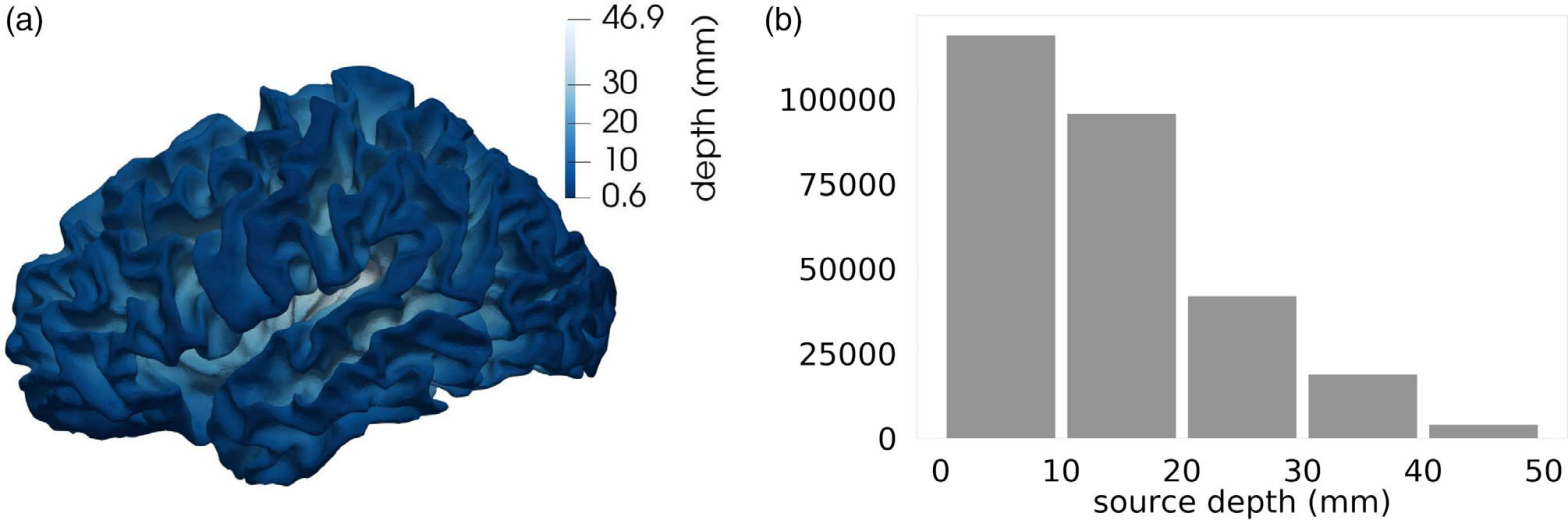
(a) Source depth with respect to the closest nodes on the inner skull surface mesh visualized on the cortical mantle. (b) Histogram of cortical source depth represented in five bins

**FIGURE 4:**
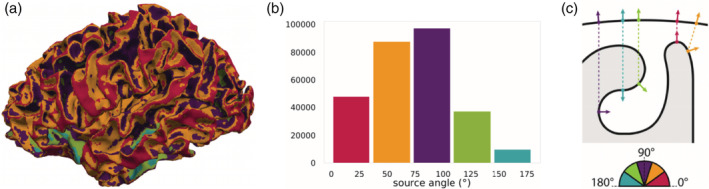
(a) Source angles with respect to the closest nodes on the inner skull surface mesh visualized on the cortical mantle. (b) Histogram of cortical source angles represented in five bins. (c) Schematic representation of the cortical and inner skull surfaces (black curves) with examples of one dipole for each bin of the angle histogram. The color‐coding guides the association in all three subfigures

In order to quantify the influence of dipole depth and orientation on the cortical SNR measures, we introduced two metrics, similarly to previous studies, for example, (Haueisen et al., 2012; Hunold et al., 2016). More precisely, the depth of each cortical source was determined by the Euclidean distance to the closest node lying on the surface mesh of the inner skull, that is, the surface mesh separating the CSF and the skull compartment. Furthermore, the angle between the cortical dipole orientation and the normal of the closest node on the inner skull surface was computed. In addition, we clustered source depths and angles into five bins to enable a quantitative overview of the cortical SNR results, in addition to the SNR maps. The source depths and source angles, with the relative histograms, can be seen in Figures 3 and 4.

We can interpret the sources in the first and last bin of the source angle histogram in Figure 4b as radial sources, the sources belonging to the central bin as tangential sources, and the remaining sources have mixed orientations, that is, orientations in‐between radial and tangential.

In Figure 4c, a schematic representation of the cortical and inner skull surfaces, in black, is depicted, together with an example of one dipole for each bin of the angle histogram (Figure 4b).

#### 2.4.2 Singular value decomposition of MEG subcortical results

As discussed above, in most of the subcortical regions there is no preferred orientation of the sources. Furthermore, it is well‐known that radial sources do not contribute to the magnetic field measured outside of a spherical volume conductor model (Sarvas, 1987). When dealing with realistically shaped head models, this still holds, in an attenuated form such as the singular values for radial direction sources are remarkably smaller than the ones for the two tangential directions in MEG leadfields. For this reason, following (Huang et al., 2007), a singular value decomposition (SVD) of MEG forward solutions was performed to identify radial and tangential components of MEG and EEG solutions. More precisely, for each subcortical source space node *i*, the corresponding MEG leadfield *L* has the dimension *n* × 3, with *n* the number of MEG channels and the three Cartesian source directions. When applying a SVD, *L* = *USV*^*t*^, the third column of *V* is the eigenvector corresponding to the smallest singular value (third diagonal entry in *S*). The latter represents well the weak contribution of a radially oriented source at source space node *i* to the MEG field, whereas the first two singular values in *S* indicate the much larger contribution of two dipoles in the tangential plane to the MEG.

The MEG and, consequently, the EEG leadfield are then projected accordingly and the radial and two tangential components of the solutions are assigned.

Note that for sources belonging to a subcortical volume, the definition of orientation and depth used for cortical sources is more ambiguous. In particular, the projection of deep sources onto a reference surface mesh is difficult to justify (Attal et al., 2012). We therefore opted here for the SVD analysis.

### 2.5 EEG and MEG forward solutions

The EEG and MEG forward problems, derived from the quasi‐static approximation of Maxwell's equations (Brette & Destexhe, 2012; Hämäläinen et al., 1993), were solved applying an FEM with Lagrangian basis functions and the so‐called partial integration source modeling approach (Pursiainen, Lucka, & Wolters, 2012; Weinstein, Zhukov, & Johnson, 2000; Yan, Nunez, & Hart, 1991). Moreover, the transfer matrix method was used to reduce the computational effort (Gençer & Acar, 2004; Piastra et al., 2018).

The code used in the simulations is implemented in the DUNEuro software (Nüßing et al., 2019) and validated in Nüßing, Wolters, Brinck, and Engwer (2016), Engwer, Vorwerk, Ludewig, and Wolters (2017), and Piastra et al. (2018). Example scripts to compute the EEG and MEG forward problems are available on GitLab (https://gitlab.dune-project.org/duneuro/duneuro-py/snippets) and at the DUNEuro website (http://duneuro.org/).

### 2.6 SNR mappings

We computed SNR mappings to cortical and subcortical dipolar sources and for both EEG and MEG. We analyzed their sensitivity to three different head volume conductor models, described in the sections above. We adopted the SNR definition of (Goldenholz et al., 2009) for our EEG/MEG sensitivity analysis:

(1) $$\mathrm{SN}R^{i}=10\mathrm{lo}g_{10}\left(\frac{{\left(a^{i}\right)}^{2}}{N}\sum_{k=1}^{N}\frac{{\left(b_{k}^{i}\right)}^{2}}{s_{k}^{2}}\right)$$

for each point‐like dipolar source *i*, where *a*^*i*^ is the source amplitude (i.e., 10 nAm, as suggested in Hämäläinen et al. (1993) and Goldenholz et al. (2009)), *N* is the number of sensors (i.e., 271 coils and 71 electrodes, after rejection of bad channels), $b_{k}^{i}$ is the EEG (in μV) or MEG (in fT) forward solution at sensor *k*, and $s_{k}^{2}$ is the noise variance at sensor *k*, deduced from the pretrigger baseline interval of the combined EEG/MEG recordings from Section 2.2, as suggested in Goldenholz et al., 2009]. More specifically, $s_{k}^{2}$ is in the order of magnitude of 10 μV^2^ and 1*e*4 fT^2^ for EEG and MEG, respectively. In addition, we computed the so‐called *differential SNR maps* related to cortical sources, following the formula

(2) $$D^{i}=\mathrm{SN}R_{\mathrm{MEG}}^{i}-\mathrm{SN}R_{\mathrm{EEG}}^{i},$$

for each dipole *i*.

### 2.7 Visualization of the results

Inflated surfaces were produced in MeshLab starting from the cortical mantle and used to visualize (with *ParaView* (http://www.paraview.org/) the differential SNR maps (see Equation (2)) related to cortical sources when the 3CI, 4CI, or 6CA head model was used. We used boxplots and heat maps to visualize the remainder of the results.

## 3 RESULTS

The presentation of results is split between cortical and subcortical sources.

**FIGURE 5:**
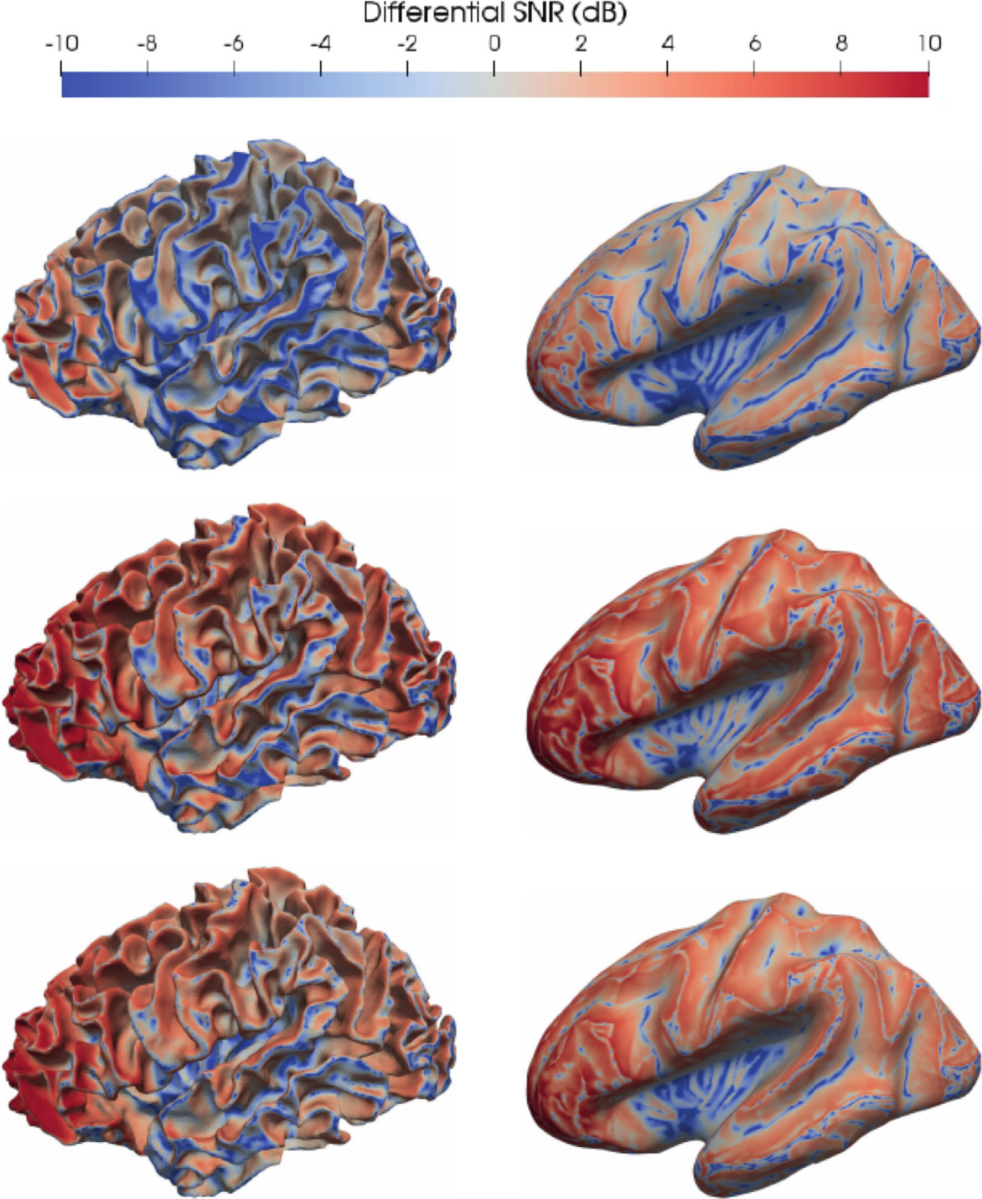
Differential signal‐to‐noise ratio (SNR) when using head model 3CI (first row), 4CI (second row) and 6CA (third row) visualized on the cortical source space (left column) and on the inflated cortical source space (right column). The values are expressed in decibels. The areas depicted in red are the areas where the SNR of the magnetoencephalography (MEG) is larger than the SNR of the electroencephalography (EEG) and the areas depicted in blue are the areas where the SNR of the EEG is larger than the SNR of the MEG. The same scaling from −10 to +10 dB is used for all head models to enable an easier comparison

In Figure 5, we computed the differential SNR presented in Formula (2) for cortical sources and for each of the three models, that is, 3CI (top row), 4CI (middle row), and 6CA (bottom row), on both the original cortical source space (left column) and the inflated cortical source space (right column). The areas depicted in red are the areas where the SNR of the MEG is larger than the SNR of the EEG and the areas depicted in blue are the areas where the SNR of the EEG is larger than the SNR of the MEG. The same scaling from −10 to +10 dB is used for all head models to enable an easier comparison.

In all three models, we can observe that the SNR of EEG is larger at the gyri crowns and sulcal valleys, where the orientations of the sources are rather radial. In a complementary way, the SNR of MEG is larger at the sulcal walls, where the orientation of the sources is mainly tangential.

When comparing the maps throughout the models, we can observe that the areas where the SNR of EEG is larger are reduced when the number of compartments included in the model is increased, especially when including the CSF compartment. Furthermore, when increasing the number of compartments, the areas where the SNR of MEG are larger do not only grow in size (more red in middle and lower rows), but also the difference between the SNRs of both modalities increases (darker red in middle and lower rows). The CSF thus weakens the sensitivity profile of EEG when compared to the one of MEG. The distribution of the differential SNR between both modalities with respect to the distinction between gyri and sulci is highlighted by the inflation of the cortical source space (right column in Figure 5). MEG SNR values are particularly high in frontal areas, which may be due to the better coverage of frontal areas by the MEG.

**FIGURE 6:**
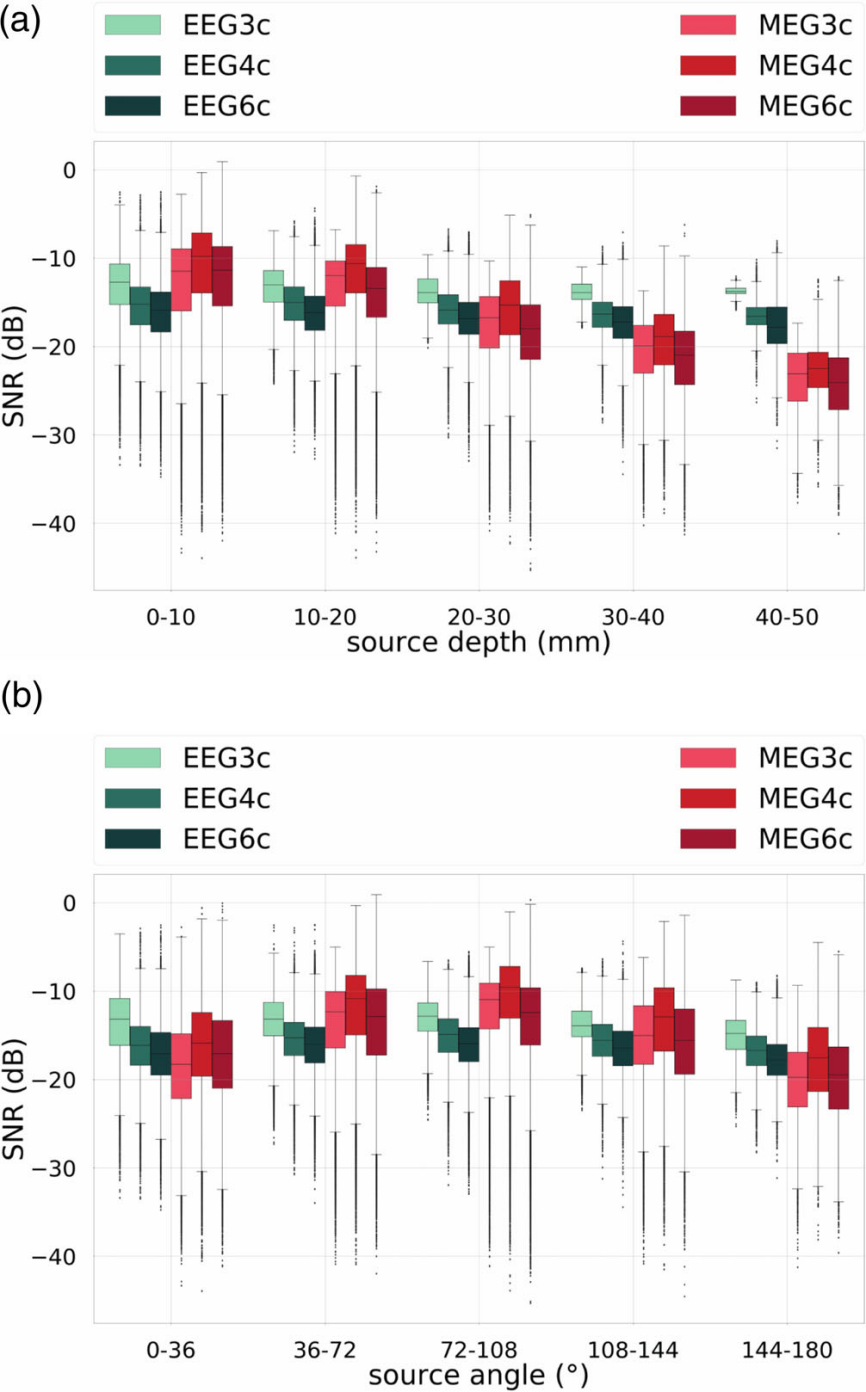
Signal‐to‐noise ratio (SNR) values for cortical dipoles for electroencephalography (EEG) (in green shades) and magnetoencephalography (MEG) (in red shades) sorted by (a) source depth and (b) source angle for the three‐compartment isotropic (3CI), four‐compartment (+CSF) isotropic (4CI) and six‐compartment anisotropic (6CA) head models. In the boxplots, the analysis includes maximum and minimum, indicated by upper and lower error bars, and thereby the total range. Furthermore, it includes the interval between upper and lower quartile, that is, the interquartile range, which is marked by a box with a black dash showing the median

As a further study, we investigated the modulation of SNR cortical values by source depth and source orientation. In Figure 6, boxplots of SNR values sorted by increasing source depth (upper subfigure, x‐axis) and angle (lower subfigure, x‐axis) are reported.

From Figure 6, we notice that EEG SNR values are mainly constant throughout varying source depth and angle. This is not the case for MEG. In Figure 6a, it is indeed noticeable that the more superficial sources are, the more MEG SNR values are higher than EEG SNR values. This trend gradually reverses when increasing the depth of the sources, until the last bin, that is, depth of 40–50 mm, where the situation is the opposite: EEG SNR values are higher than MEG SNR values. From Figure 6b, we observe that MEG SNR values are higher for more tangential sources, that is, sources corresponding to the central bin, and lower for more radial sources, that is, sources in the first and last bin.

**FIGURE 7:**
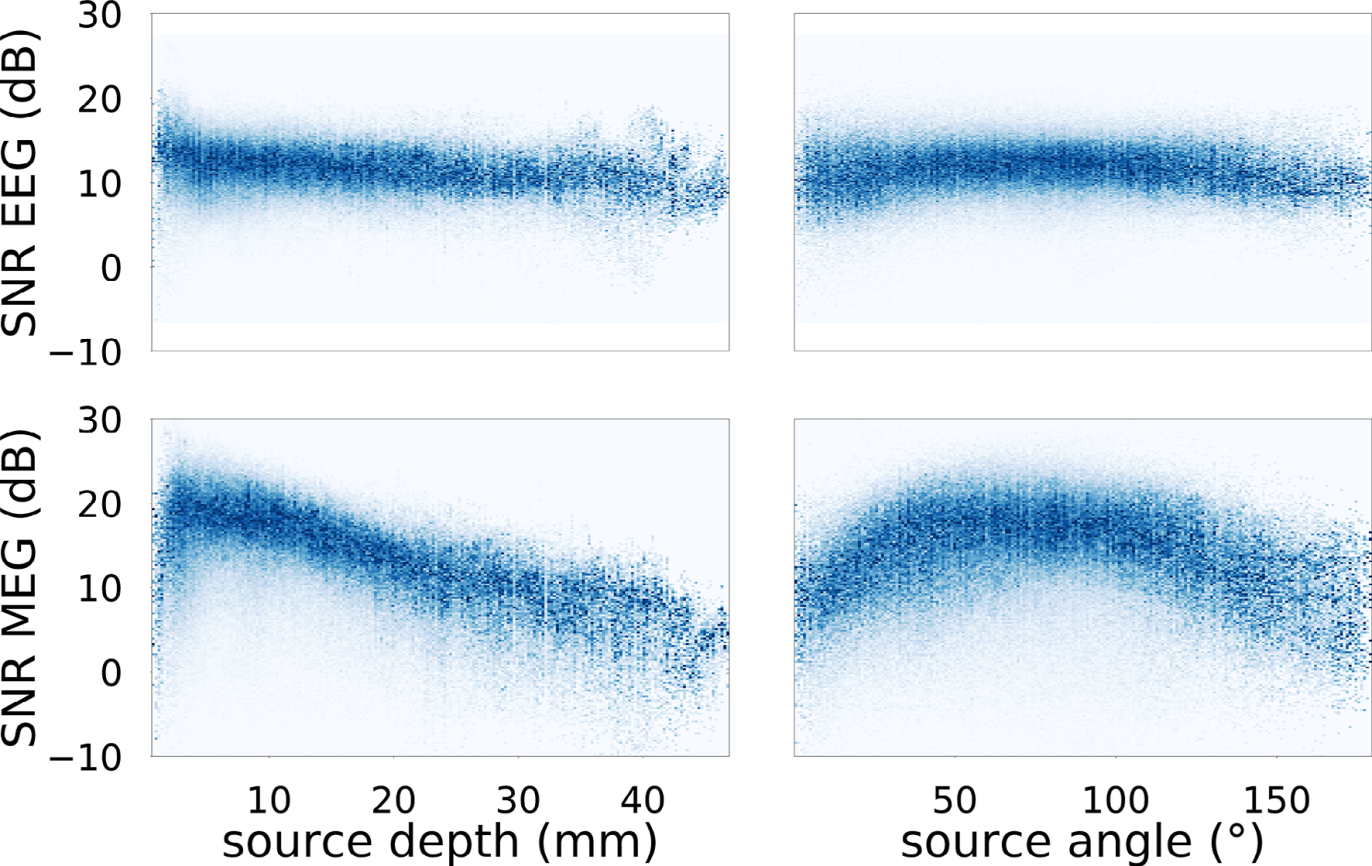
Heatmaps normalized by column of electroencephalography (EEG) (upper row) and magnetoencephalography (MEG) (lower row) signal‐to‐noise ratio (SNR) values in dependency of source depth (left) and source angle (right) from the inner skull surface, when 6CA was adopted. Only cortical sources are taken into consideration here

In order to further investigate the modulation of SNR values by source depth and orientation, we created heat maps, that is, bidimensional histograms, normalized by column for EEG and MEG SNR values with respect to source depth and angle, when 6CA was adopted (see Figure 7).

From Figure 7, a weak modulation of source depth and orientation for EEG SNR values is confirmed. We notice a slow SNR decrease for increasing depth. For MEG SNR values, the behavior is quite different. In Figure 7 (lower left), we observe how SNR values are extremely low for very superficial sources, but they increase to reach their maximum within a few mm. After the peak, SNR values decrease with source depth in a stronger way than for the EEG. Note the scatter of SNR values for depths and angles where the fewest sources are (see histograms in Figures 3b and 4b), that is, depths larger than ~30 mm and angles smaller than 25° and larger than 150°.

**FIGURE 8:**
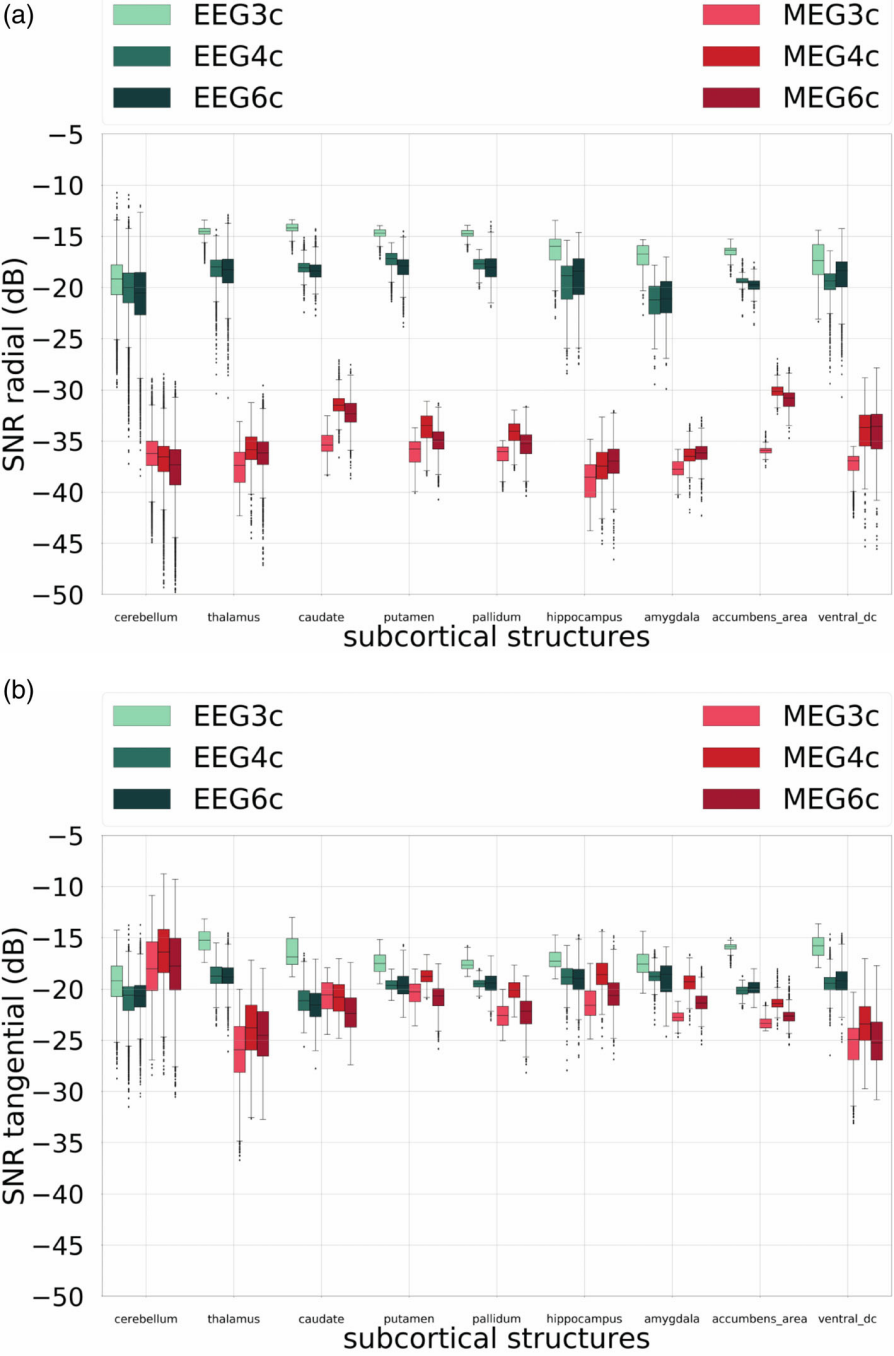
Signal‐to‐noise ratio (SNR) values for radial (a) and the strongest tangential (b) components of electroencephalography (EEG) and magnetoencephalography (MEG) for subcortical dipoles. On the x‐axis, the nine subcortical areas considered are listed, on the y‐axis, the correspondent SNR values for head models 3CI, 4CI, and 6CA. Cold colors are used for the EEG SNR values, warm colors for the MEG SNR values. In the boxplots, the analysis includes maximum and minimum, indicated by upper and lower error bars, and thereby the total range. Furthermore, it includes the interval between upper and lower quartile, that is, the interquartile range, which is marked by a box with a black dash showing the median

For the visualization of SNR values for subcortical dipoles, we used boxplots, as, in this case, we are dealing with sources lying in a volume and not on a surface. In Figure 8, the SNR values for EEG and MEG, and for all three head models are shown when considering the radial (Figure 8a) and a tangential (Figure 8b) component of the EEG and MEG. The behavior of the SNR values of the tangential orientation related to the second highest singular value in the SVD decomposition was very similar and therefore omitted here.

From Figure 8a, we can notice that, first of all, the EEG SNR values for the subcortical radial sources are systematically much larger than the MEG SNR values. The differences between median values are between about 10 and 20 dB. Second, the difference between results of head models 4CI and 6CA is small compared to the difference between 3CI and 4CI/6CA, and the latter is the case for all subcortical areas. Third, the EEG SNR values for three compartments are larger than when four or six compartments are considered. The opposite is shown for the MEG SNR values: the SNR values are lower when the 3CI head model is used in all subcortical areas but the cerebellum, where there seems to be no remarkable difference between the three models.

Finally, for tangential sources, in Figure 8b, we do not notice a clear difference between EEG and MEG. It is indeed remarkable that the range of MEG SNR values coincides with the one of the EEG SNR values. Whereas for cerebellum, the MEG SNR is even larger than the EEG SNR, it is the inverse for the thalamus and for all other subcortical structures, where EEG and MEG results in more or less similar SNR values.

## 4 DISCUSSION

In this work, we present a comprehensive study on EEG and MEG sensitivity to cortical and subcortical sources. We aimed at making available new up‐to‐date sensitivity simulation results in a unified and state‐of‐the art framework using detailed FEM head models.

Experimental (Rice et al., 2013) and simulation studies (Vorwerk et al., 2014) have already shown that CSF modeling has a considerable influence on EEG forward solutions. Indeed, when such a conductive material surrounding the brain is included in the model, a shunting phenomenon occurs and leads to a decrease of the EEG signal amplitude. Nevertheless, the sensitivity maps and studies presented so far in literature do not take these effects into consideration, possibly leading to inaccurate results and conclusions. In Goldenholz et al. (2009), for example, EEG and MEG SNR maps were computed, compared and visualized in inflated cortical mantles and their SNR formula is also the one we adopted for our study (see Equation (2)). However, Goldenholz et al. (2009) performed BEM computations in a three‐compartment isotropic head model, thus, ignoring the EEG weakening effect of the CSF compartment. The EEG SNR overestimation is therefore present in their differential SNR map, leading to the conclusion that the cortical strips where MEG is less sensitive than EEG are even thinner, as shown by our results. Also in Hunold et al. (2016), BEM is used in a three‐compartment head model. The EEG SNR values reported in their work are therefore also overestimated.

In our study, we do not only clearly reproduce the high CSF effects on EEG SNR values, but we provide new SNR mappings in highly detailed head models for drawing conclusions on source reconstruction studies, or, in a reciprocal way, for optimizing brain stimulation set‐ups, and, more generally, for the designing of new bioelectromagnetism experiments. A further novelty of this study is that such sensitivity estimations are additionally computed for subcortical sources, where, for the first time, evidence about the importance of modeling the CSF also in this scenario is provided.

It is crucial to take into account that a main consequence of our result for the EEG SNR maps is that MEG might be, all in all, more sensitive to the majority of cortical sources than EEG. This aspect was less evident in previous studies (Goldenholz et al., 2009; Hunold et al., 2016). These results are in agreement with simultaneous EEG/MEG measurements in epilepsy. For example, in Iwasaki et al. (2005), from 43 epilepsy patients, interictal spikes were captured in MEG alone in eight (18%), in EEG alone in one (2%), and the combined sensitivity was 31/43 patients (72%). In Knake et al., 2006), MEG‐only interictal epileptiform discharges (IED) were recorded in 9 out of 67 patients (13%), whereas in only two EEG‐only IEDs were recorded (3%) and the combined sensitivity was 50/67 patients (75%). Furthermore, for example, in SEP and SEF studies, it was shown that while the radial P22 is mainly only measurable in EEG, the SNR of the superficial and more tangentially oriented P20 and N30 components are much higher in MEG than EEG (Allison, Wood, McCarthy, & Spencer, 1991; Antonakakis et al., 2020; Aydin et al., 2017; Buchner et al., 1994; Fuchs et al., 1998; Papadelis, Eickhoff, Zilles, & Ioannides, 2011). The majority of cortical sources is thus rather tangentially oriented and therefore more visible in MEG, in agreement with the results of our Figure 5. On the other side, deep sources such as those underlying the SEP/SEF 14 ms component (Fuchs et al., 1998) or auditory brainstem responses (Parkkonen, Fujiki, & Mäkelä, 2009) are, however, better detectable by EEG than by MEG.

Moreover, in additional investigations (not shown here), we did not find the spatial sampling of the MEG sensors to have an influence on our results, whereas the spatial coverage is important. In these investigations, we computed SNR values for 68 regularly distributed MEG sensors, instead of 271, and all the 71 EEG sensors, and we found hardly any difference with regard to our main conclusions.

Regarding the MEG case, Hillebrand and Barnes (2002) and Attal et al. (2012) conducted MEG sensitivity studies and computed MEG sensitivity maps. In both cases, they focused on MEG only and they used simplified head models and methods to compute the forward solutions. While the main conclusions are in line with our findings, they lack in relating the MEG SNR maps to the EEG SNR maps, one of the main objectives of our comprehensive study. We have indeed used the same highly detailed head models for EEG and MEG forward computations.

As previously already observed, electric and magnetic sensitivity maps have to be taken into account when evaluating data in neuroscientific and neurodiagnostic applications not only in EEG and MEG source analysis, but also in TES or TMS. In brain stimulation research the EEG and MEG sensitivity maps produced in this work can be indeed taken into account in a reciprocal way (Vallaghé et al., 2008; Wagner, Burger, & Wolters, 2016), guiding the optimization of the stimulation configuration (Guler et al., 2016; Sadleir et al., 2012; Wagner, Lucka, et al., 2016).

In the current implementation, we modeled EEG/MEG sensors as point‐sensors. While more realistic models like the complete electrode model for EEG (He, Rezaei, & Pursiainen, 2019; Ollikainen, Vauhkonen, Karjalainen, & Kaipio, 2000; Pursiainen, Agsten, Wagner, & Wolters, 2017) and higher order integration for MEG (Roth & Sato, 1993) forward modeling exist, it was shown for the EEG in Pursiainen et al. (2012) (and even for a neonate with large sensor to head surface ratio (Pursiainen, Lew, & Wolters, 2017) and for the MEG in Dachwitz (2019) that these modeling improvements will not considerably influence our main conclusions here.

In reality, there is a large inter‐subject variability in CSF volume (section 12.3.4 in Benninghoff (2004)), with highest volumes more than 60% larger than lowest ones in healthy subjects. Abnormalities such as brain atrophy (e.g., in dementia, Alzheimer, or anorexia), stroke lesions or hydrocephalus might correspond to a much‐increased intracranial fluid volume (Sakka, Coll, & Chazal, 2011). It is therefore important to compute individual sensitivity maps. Furthermore, the compartment between brain and skull, homogenized in this study as the CSF compartment, contains also other tissues such as dura mater and blood vessels. We assumed for CSF a fixed conductivity value of 1.79 S/m due to the study of (Baumann et al., 1997), who measured this average over seven CSF samples at body temperature with less than 2% deviation across a frequency range of 10 Hz to 10 kHz. Even if this is nearly identical to the recommended weighted average mean value of 1.71 S/m of a recent meta‐analysis (McCann, Pisano, & Beltrachini, 2019), a larger variability of CSF conductivity was reported when using magnetic resonance electrical impedance tomography instead of direct applied current for its determination (Figure 8 in McCann et al. (2019)). These aspects might reduce the sensitivity of the EEG to the CSF and the EEG SNR overestimation discussed for the three‐compartment head model. On the other side, especially in pathological situations such as in brain atrophy, stroke lesions or hydrocephalus, the described CSF shunting effect might be much larger than presented here. Therefore, individualized SNR maps should be drawn using head models that also include dura mater (Ramon, Garguilo, Fridgeirsson, & Haueisen, 2014) and blood vessels (Fiederer et al., 2016).

Also individual skull conductivities (Antonakakis et al., 2020) and local skull inhomogeneities such as sutures, which could provide a path of higher conductance (Ollikainen et al., 2000; Pohlmeier et al., 1997; Tang et al., 2008), might have to be taken into account. Especially in neonates (Azizollahi et al., 2016) and infants (Lew et al., 2013), the inclusion of skull conductivity inhomogeneities can have high importance and should therefore not be neglected.

Due to our noise estimation procedure from measured SEP and SEF data, we only investigated sensitivity maps for our EEG and MEG (axial gradiometers) systems. However, not only the individual head model influences the sensitivity maps, as discussed here, but also the specific systems (see, e.g.,Wendel et al., 2009). Future investigations should thus be carried out to compute and compare sensitivity maps between different EEG and MEG (e.g., axial vs. planar gradiometers vs. magnetometers) systems.

Finally, as a follow‐up project, it might be interesting to use the SNR maps created in this study to weight the leadfield matrices and investigate the influence on the accuracy of combined EEG/MEG source reconstruction results, similarly to Fuchs et al. (1998), Muravchik and Nehorai (2001). In Muravchik and Nehorai (2001), they indeed used statistical methods to relate the SNR to source localization errors. Furthermore, this study can be extended in order to create and study sensitivity maps for neonates (Mahmoudzadeh, Wallois, Kongolo, Goudjil, & Dehaene‐Lambertz, 2017; Roche‐Labarbe et al., 2008), children (Aarabi, Kazemi, Grebe, Moghaddam, & Wallois, 2009) or patients with brain lesions (Datta, Baker, Bikson, & Fridriksson, 2011; Minjoli et al., 2017), together with different sensor configurations, for example, intracranial EEG sensors (Branco et al., 2018).

## 5 CONCLUSIONS

In this work, we computed and analyzed EEG and MEG SNR mappings for three head models, from an isotropic three‐compartment head model, to an isotropic four‐compartment head model and a detailed six‐compartment head model with anisotropic white matter using state‐of‐the‐art FEM computations. We used two source spaces, namely a cortical surface and a subcortical volume.

Our study had two main goals. First, we assessed the impact of forward modeling accuracy on sensitivity maps. Second, we extracted useful and novel insights from our EEG and MEG sensitivity maps.

Results show that, first, a three‐compartment head model leads to overestimated EEG SNR values for both cortical and subcortical sources. We therefore made available sensitivity maps which are built on a more detailed head model that also includes a representation of the highly conducting CSF compartment. Second, we conclude that MEG is more sensitive than EEG to the majority of cortical sources. Finally, as to subcortical sources, our results show that MEG is not blind for deep tangential sources.

Our comprehensive sensitivity guideline could encourage brain researchers and clinicians to use combined EEG/MEG or, if a combination is not feasible, the choice of either EEG or MEG modality for their experimental setup or diagnosis. Furthermore, our results might guide a correct interpretation of neuroscientific and neurodiagnostic applications such as EEG/MEG source localization or, in a reciprocal sense, TES/TMS sensor placement optimization.

## CONFLICT OF INTEREST

The authors declare no conflict of interest.

## Data Availability

The three volumetric meshes and the neurophysiological data are available on the Zenodo portal (Piastra et al., 2020). Script examples to compute the EEG/MEG forward solutions can be found on GitLab (https://gitlab.dune-project.org/duneuro/duneuro-py/snippets) or on the DUNEuro webpage (http://duneuro.org/). The raw MRI scans are not publicly available due to privacy or ethical restrictions.
